# Burden of influenza, healthcare seeking behaviour and hygiene measures during the A(H1N1)2009 pandemic in France: a population based study

**DOI:** 10.1186/1471-2458-12-947

**Published:** 2012-11-05

**Authors:** Dieter Van Cauteren, Sophie Vaux, Henriette de Valk, Yann Le Strat, Véronique Vaillant, Daniel Lévy-Bruhl

**Affiliations:** 1 Department of infectious diseases. Institut de Veille Sanitaire (InVS) (French Institute for Public Health Surveillance), 12 rue de Val d’Osne, St Maurice Cedex, 94415, France

**Keywords:** Self-defined influenza, Burden, Epidemiology, Incidence, Surveillance

## Abstract

**Background:**

Influenza surveillance systems do not allow the identification of the true burden of illness caused by influenza in the community because they are restricted to consulting cases. A study was conducted to estimate the incidence and the burden of self-defined influenza, and to describe healthcare seeking behavior for self-defined influenza during the A(H1N1)2009 pandemic in the French population.

**Methods:**

We conducted a random-based retrospective cross-sectional telephone survey between May 2009 and April 2010 among a random sample of the French population.

**Results:**

For the 10 076 people included, 107 episodes of self-defined influenza were reported. The annual incidence of self-defined influenza was estimated at 13 942 cases per 100 000 inhabitants (CI95% 10 947 – 16 961), 62.1% (CI95% 50.5 – 72.5) of cases consulted a physician and 11.3% (CI95% 5.5 - 21.7) used a face mask. Following recommendations, 37.5% (CI95% 35.5 – 39.5) of people in the survey reported washing their hands more often during the pandemic season, and there was a positive association with being vaccinated against A(H1N1)2009 influenza, being a women, being a child (< 15 years) or living in a big city (≥ 100 000 inhabitants).

**Conclusions:**

Self-defined influenza causes a significant burden of illness in the French population and is a frequent cause for consultation. These results allow a more accurate interpretation of influenza surveillance data and an opportunity to adapt future health education messages.

## Background

In France as in other countries, influenza surveillance systems are mainly based on data collected from physicians who report cases matching the case definition. Data given by these sentinel networks allow detection of the start and end of influenza epidemics. However, the results of this surveillance do not allow the identification of the true burden of illness caused by influenza in the community because they are restricted to consulting cases. Data from the literature indicate that up to 60% of individuals with influenza do not visit physician and this proportion differs between countries, reflecting differences in the healthcare systems and socio-cultural differences in healthcare seeking behaviours [1]. Moreover this proportion can vary from one season to the next depending on the circulating strain [1-3]. On 11 June 2009, the World Health Organization (WHO) declared a pandemic due to the novel A(H1N1)2009 influenza virus [4]. During a pandemic, the changes in healthcare utilisation in comparison with seasonal influenza epidemics are unpredictable. Because of the anxiety in the population or the overflow of healthcare services, for instance, data produced by sentinel networks could induce an over or an underestimation of the true burden of influenza. As part as an ongoing community study on the burden of seasonal influenza, the French Institute for Public Health Surveillance (InVS) conducted a national population based study between May 2009 and April 2010, in order to estimate the incidence and the burden of self-defined influenza, and to describe healthcare seeking behaviour for influenza during the A(H1N1)2009 pandemic in the French population.

## Methods

### Study population and sample

We carried out a retrospective cross-sectional telephone survey between May 2009 and April 2010 among a random sample of the French population. The French overseas departments (Guyana, Antilles, Reunion Island) were not included in this study.

The study population included all people living in residential households connected to a land telephone line and who spoke French. Households and household members were randomly selected for interview. At the first stage, the sampling frame was the French mainland telephone directory stratified by region and town size. Each month a list of 2800 numbers was selected randomly from the French telephone directory. Each number was then incremented by one, in order to generate a list that also included unlisted telephone numbers. At least 20 attempts were made at different times of the day (between 16:00 and 21:00 hours during the week and between 10:00 and 14:00 hours on Saturdays) before a phone number was abandoned. All non-residential telephone subscribers, such as offices, institutions or holiday homes, were excluded from the study.

At the second stage, one person aged ≥ 5 years and one child < 5 years (if any) were randomly selected among the household members by selecting the person who had the next birthday. If the selected person was aged between ≥ 12 and < 18 years, a parent could choose to answer for the child or allow the child to answer. If the child was < 12 years old, one parent was asked to answer on the child’s behalf.

All interviews were conducted by professional interviewers, using Computer Assisted Telephone Interviewing (CATI). The interviewers were monitored by supervisors (ratio 6:1). Daily quality controls were performed by supervisors. A pilot study was conducted in March-April 2009 (169 interviews). The survey had two main outcomes: to obtain accurate estimates of the incidence and burden of self-defined influenza and acute gastroenteritis (AG) [5]. The sample size of 9600 (800 per month) took into account these two outcomes. An expected design effect of 2 has been taken into account for the 10% expected households where one adult and one child would be interviewed. This sample size allowed an annual precision of 0.25% for a significance level of 5% for self-defined influenza as the expected 4-week incidence was 1.5% for influenza [2] (http://sentiweb.org/).

### Data collection

Cases of self-defined influenza were defined as having influenza (“flu”) with onset of symptoms within the four weeks before the interview. A seven-day symptom-free interval was defined to distinguish multiple episodes.

The sex and age of each respondent were collected, as well as socio-demographic characteristics of the household: household size and age of people living in the household, education level and occupation of the head of the household.

Self-defined flu cases were asked questions about symptoms, duration of illness, illness in other household members, use of healthcare services, diagnostic methods and treatment practices. In the case of multiple episodes, only the most recent episode of self-defined influenza was described. Questions related to the implementation of personal hygiene control measures were asked of cases older than 14 years. If cases were aged 20 – 64 years, they were asked whether they were healthcare workers.

A predefined questionnaire with the same set of questions was used throughout the study. An additional question was implemented in January 2010 to estimate the impact of the communication campaign for pandemic influenza on handwashing habits. According to national regulations, ethical approval was not required for this observational retrospective study [6]. However, a verbal consent was obtained for the interview and all data transmitted to InVS were anonymous.

### Statistical analysis

All estimates took into account the sampling design components (primary sampling unit, sampling weights). For each respondent, sampling weights were adjusted by age, sex, region, household size and size of town population. The 4-week incidence was calculated by dividing the number of episodes of self-defined influenza with onset of symptoms within the four weeks prior to the interview by the total number of respondents for that time period.

Weekly estimated incidences from the French Sentinel Network (http://sentiweb.org/), composed of general practitioners, were used to compare the estimated incidence of consultation for self-defined flu of this study with the estimated incidence of consultation for influenza-like illness (ILI) from the network (the four week period before the last day of interview was taken as reference). The case definition of ILI in this network is the sudden appearance of fever and myalgia associated with respiratory symptoms. Except for the suddenness, we used an identical symptom based case definition in order to compare the estimations of our study with the estimations from this network. Because of the difficulty for children of reporting myalgia, we considered that children younger than 15 years had ILI if they reported respiratory symptoms associated with fever or myalgia. Estimates of medical consultations for ILI take into account the estimate of French population (Insee, 2009).

Possible determinants of the implementation of the recommendations to prevent A(H1N1)2009 transmission were investigated using univariate and multivariable logistic regressions. Explanatory variables tested were: age, sex, presence of children aged <5 years and number of people in the household, size of town population, being a case of self defined influenza, being an at-risk individual for seasonal influenza complications (defined as a person who reported having received a personal voucher for free seasonal vaccination from the national health insurance fund), vaccination against A(H1N1)2009 influenza and occupation of the head of the family. Symptoms and duration of illness were additional explanatory variables tested for healthcare seeking behaviour. All variables were introduced into the multivariable model. A global P value was calculated for categorical variables (Wald’s test). The final multivariable model was built using backwards elimination. Only age, sex and variables with P <0.05 were kept in the final model. Odds ratios, adjusted odds ratios and 95% confidence intervals (95% CI) are presented for the main findings.

Interaction effects and collinearity between variables were tested. To assess whether any variables in the final model were subject to confounding by any variables that had been omitted from the final model, each omitted variable was re-introduced individually and tested for significance. Confounding was determined by looking for a change of ≥30% in regression coefficients. Data analyses were performed using Stata 9.2® (StataCorp, USA).

## Results

### Response rate

Of the 32 676 phone numbers selected, contact was established with 17 036 (52.1%); 1053 phone numbers were excluded because they did not correspond to a residential household. Of the 15 983 households eligible for the survey, 8905 agreed to participate (response rate: 55.7%). Reasons for refusals (more than one response possible) were: “lack of time” (42%), “not interested in the survey” (42%) and “never answer interviews” (21%). Each month, approximately 750 households (825 participants) were included and the response rate was stable throughout the entire study period. From the 10 130 people randomly selected within these 8905 households, 10 076 people were included in the survey (99%). The sample was representative in terms of gender, region, town size, and age (with the exception that children under five years of age were overrepresented due to the survey method).

### Estimated incidence of influenza

**Figure 1:**
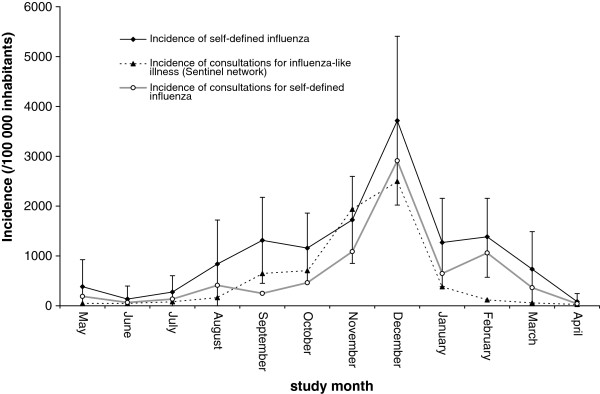
**Incidence of self-defined influenza,consultations for influenza-like illness(Sentinel system)and consultations for self-defined influenza by study month,France,May 2009 to April 2010.**

Of the 10 076 people included in the study, 105 people reported 107 episodes of “flu” within the four weeks prior to the interview. The annual incidence rate of self-defined influenza was estimated at 13 942 cases per 100 000 inhabitants (CI95% 10 947 – 16 961) which represents more than 8.7 million episodes of self defined influenza in France in 2009–2010. Taking into account the symptoms described, 71 episodes of self defined influenza (66.5%) met the ILI case definition, suggesting 5.5 million episodes of ILI occurred in France in 2009–2010. Trends of monthly incidence of consultations for self-defined influenza estimated in our survey are comparable with the monthly incidence of consultations for ILI given by the sentinel system (Figure 1). Incidence peaks were observed in December 2009 with both curves.

### Description of cases

**Figure 2:**
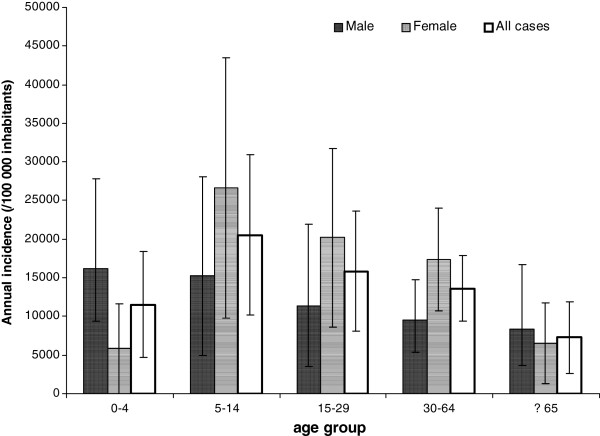
**Incidence of self-defined influenza by sex and age group,France,May 2009 to April 2010.**

Incidence of self-defined influenza was highest in the 5–14 year age group (20 548 cases/100 000, CI95% 10 166–30 930), and lowest among people older than 64 years (7 279 cases/100 000, CI95% 2 618 – 11 940) (Figure 2). No statistical differences in incidence by sex were observed among cases. Cases were significantly younger than individuals who did not report influenza illness (32.1 vs. 39.6 years, p<10^-3^).

It was estimated that 64.8% (CI 95% 54.0 – 74.3%) of the cases were not symptomatic anymore at the time of the interview. The mean duration of illness of these cases was 6.7 days (CI 95% 5.4 – 8.0 days).

Cough (85.7%) and asthenia (82.8%) were the most frequently reported symptoms, followed by headache, nasal congestion and fever (Table 1). Twenty seven per cent reported concomitant gastrointestinal symptoms (defined as diarrhoea or vomiting/nausea). These cases were not statistically different regarding age, sex and season from cases that did not report concomitant gastrointestinal symptoms.

**Table 1:** **Symptoms of self defined influenza**, **France**, **May 2009 to April 2010** (**n**=**105**)

**Symptoms**	**Proportion**	**CI 95%**	
Cough	85.7	77.2	91.4
Asthenia	82.8	73.0	89.5
Headache	78.2	68.7	85.4
Nasal congestion, sneezing	76.3	65.7	84.3
Fever	75.0	64.4	83.3
Feverishness	73.0	62.6	81.4
Sore throat	71.2	61.0	79.7
Myalgias	70.1	59.6	78.8
Dyspnea	40.7	30.6	51.6
Chest pain	36.5	26.7	47.5
Gastrointestinal	27.0	18.1	38.1
Confusion, faintness	11.1	6.1	19.2

### Healthcare seeking behaviour

The proportion of self-defined influenza cases that consulted a physician is estimated at 62.1% (CI95% 50.5 – 72.5), usually a general practitioner (61.3%, CI95% 49.6 – 71.9). Only 0.4% of the cases went to a hospital emergency department (Table 2). It was estimated that 70.1% (CI95% 54.7 – 81.9) of the self-defined cases that met the ILI case definition consulted a physician.

**Table 2:** **Consultation for self defined influenza**, **France**, **May 2009 to April 2010** (**n**=**100**)

	**Proportion**	**95%CI**	
General practitioner - office visit	50.7	39.3	62.1
General practitioner - home visit	9.1	4.8	16.5
General practitioner - on call	2.6	0.8	8.3
Pediatrician	0.8	0.2	3.2
Hospital - Emergency Department	0.4	0.1	3.0

The estimations of the incidence and healthcare seeking behavior suggest that 5.4 (95% CI 4.3 – 6.6) million medical consultations for self-defined influenza occurred in France in 2009–2010 and among these consultations 3.8 (95% CI 2.8 – 4.9) million medical consultations were related to episodes of ILI.

The main reasons for consultation of self-defined influenza cases (more than one response possible) were: high fever (25.5%) unusual/strange symptoms (24.6%) and prolonged symptoms (21.9%). The main reasons for not consulting (more than one response possible) were: the feeling that a consultation was not necessary (15.7%), quick recovery/no serious symptoms (12.5%) and too busy (5.6%).

The mean number of consultations of self-defined influenza cases was 1.2 times (range 1–3) and the mean delay before consultation was 1.9 days (95% CI 1.3 – 2.6 days) with a median of 2 days (range 0 – 15). The consultation rate was 100% among children <5 years (12/12), 66.6% (95% CI 36.0 – 87.7%) among children aged 5–14 years, 45.7% (95% CI 21.2 – 72.4%) among people aged 15–29 years, 62.7% (95% CI 46.0 – 76.9%) among adults aged 30–64 and 60.0% (95% CI 26.7 – 86.0%) among people older than 65 years. It was not possible to carry out a multivariate analysis of the determinants of consultation for self-defined influenza because of the limited sample size for this part of the study. No case in our survey was hospitalized because of flu.

### Medication

It was estimated that 88.9% (95% CI 80.0 – 94.2%) of flu cases used medication. Medications were bought with a doctor’s prescription for 58.7% (95% CI 47.1 - 69.5%) of the cases, 25.7% (95% CI 16.8 - 37.2%) came from the family medicine chest and 15.6% (95% CI 9.1 - 25.3%) were over-the-counter drugs. The mean duration of treatment was 5.5 days (95% CI 4.8 – 6.1 days, median: 5 days, range 1–20) and was significantly longer for cases who consulted (6.1 days vs. 4.0 days, p=0.002).

### Control measures and hygiene

It was estimated that 11.3% (95% CI 5.5 - 21.7%) of the cases older than 14 years used a face mask when they were sick. The main reasons given for not using a face mask were “I don’t see the point/why it is useful” for 34.5% of the cases, “the physician didn’t advise me to” for 16.3% and “not available” for 14.9% of the cases.

To blow their nose, 88.5% (95% CI 78.3 – 94.2%) of the cases reported having used a tissue, 3.6% (95% CI 1.1 – 11.3%) used a handkerchief and 3.9% (95% CI 1.2 - 11.9%) used both.

Approximately one out of every three cases (34.7% 95% CI 23.5 - 47.9%) reported washing their hands more often than usual while they were sick, 64.2% (95% CI 51.1 - 75.6%) of the cases washed their hands as usual and 1.0% (0.1 - 7.4%) washed their hands less often.

### Impact of the recommendations to prevent A(H1N1) transmission

From January to April 2010, 37.5% (95% CI 35.5 – 39.5%) of the population reported that they washed their hands more often this season than during the previous season because of the recommendations that were made to prevent A(H1N1) transmission.

A multivariable analysis was carried out in order to determine the factors associated with this change in handwashing (Table 3). The final multivariable model included age group, sex, being vaccinated against A(H1N1)2009 influenza and size of town population (Table 3). No variables in the final model were subject to identified confounders. Individuals living in big towns (≥100 000 inhabitants) washed their hands more often this season because of the recommendations, compared with individuals living in small towns (< 20000 inhabitants). The improvement in frequency of handwashing (because of the recommendations) was significantly higher among people vaccinated against A(H1N1)2009 influenza, among women and among children (< 15 years) compared with adults (30–64 years). Being an at-risk individual for seasonal influenza was not associated with an increase in handwashing (p=0.089).

**Table 3:** **Determinants of a higher frequency of hand washing because of A**(**H1N1**) **recommendations**, **France**, **January 2010 to April 2010**

		**Univariate**			**Multivariate**		
	**N**	**OR**	**CI 95**%	***p*** - **value**	**OR**	**CI 95**%	***p*** - **value**
**Age group**				0,001			>10^-3^
0 - 14 years	762	1,31	1,07-1,61	0,009	1,33	1,08 - 1,63	0,007
15 - 29 years	370	0,77	0,59 - 1,02	0,069	0,77	0,58 - 1,01	0,059
30 -64 years	1 550	ref	ref	ref	ref	ref	ref
65 years and more	553	1,24	1,00 - 1,54	0,047	1,22	0,98 - 1,52	0,072
**Gender**							
male	1 435	0,77	0,66 - 0,91	0,002	0,76	0,65 - 0,90	0,001
female	1 800	ref	ref	ref	ref	ref	ref
**At risk individual**							
yes	781	1,30	1,08 - 1,57	0,005			
no	2 454	ref	ref	ref			
**A**(**H1N1**)**2009 vaccination**							
yes	359	1,44	1,11 - 1,87	0,006	1,40	1,07 - 1,81	0,012
no	2 875	ref	ref	ref	ref	ref	ref
**Self defined flu case**							
yes	29	0,89	0,39 - 2,02	0,785			
no	3 206	ref	ref	ref			
**Presence of a child** <**5 years in the household**							
yes	470	1,13	0,90 - 1,42	0,296			
no	2 765	ref	ref	ref			
**Occupation of the head of the family**				0,015			
manual worker	594	ref	ref	ref			
farmer	58	1,10	0,15 - 8,47	0,921			
self employed	164	1,25	0,47 - 3,30	0,652			
higher professional and managerial occupation	472	0,76	0,35 - 1,64	0,486			
intermediate occupation	357	1,24	0,60 - 2,56	0,556			
clerical	563	1,30	0,65 - 2,57	0,456			
retired	903	0,37	0,17 - 0,79	0,010			
student	28	3,47	0,70 - 17,16	0,127			
unemployed	96	1,15	0,41 - 3,20	0,792			
**Town size**				0,098			0,070
< 20000 inhabitants	1 517	ref	ref	ref	ref	ref	ref
20000 - 100000 inhabitants	399	1,15	0,89 - 1,49	0,281	1,14	0,88 - 1,47	0,324
≥ 100000 inhabitants	1 319	1,21	1,01 - 1,45	0,035	1,23	1,03 - 1,48	0,022
**Household size**				0,157			
1 person	701	ref	ref	ref			
2 persons	965	0,83	0,67 - 1,02	0,076			
3 persons	574	0,83	0,65 - 1,07	0,155			
4 persons and more	995	0,99	0,79 - 1,24	0,955			

### Impact of recall period

In order to evaluate the impact of the length of the recall period, we calculated the incidence of self-defined flu with onset of symptoms within 7 days before the interview. This incidence was estimated at 17 924 cases/100 000 inhabitants (95% CI 10 988 – 24 860) and was not significantly different from the incidence estimated with a recall period of 28 days (13 942 cases / 100 000 inhabitants; p=0.14).

## Discussion

This is the first time that a population based telephone survey has been implemented to assess the burden of influenza in France. Our results suggest 8.7 million episodes and 5.4 million medical consultations of self defined influenza between May 2009 and April 2010. More than six out of ten cases consulted a physician for their illness, usually a GP. The highest consultation rate among children and the lowest among people aged 15–29 years are similar to those observed in England [7]. However, in our study these differences were not statistically significant, probably because of a lack of power.

The estimate of 3.8 million (95% CI 3.0 – 4.5) medical consultations for ILI is comparable with data produced by the sentinel system (4.2 million consultations for ILI from May 2009 to April 2010) and trends (rise, peak and decline) and estimates produced by both systems were consistent.

Symptoms reported by the self-defined flu cases in our study were compared with those of virological confirmed A(H1N1)2009 cases [8,9]. The most frequently reported symptoms such as fever and cough were very similar (86% and 92% for fever ≥ 38°C, 86% and 88% for cough). Almost nine out of ten cases used medication that was mostly bought after prescription. In a national prospective survey of household contacts carried out in France during a seasonal influenza epidemic (year 2000), the proportion of ILI cases visiting a physician was estimated at 57%, the mean number of consultations was 1.3 (± 0.6), and the proportion of medication obtained with a prescription was estimated at 90% [2]. Although the study designs of the surveys were different, the healthcare seeking behaviour for influenza observed in the context of pandemic influenza season was not significantly higher that of a “classical” seasonal influenza in France. The proportion of cases that consulted in our study was similar to Belgium (67%), but higher than the proportion observed in the Netherlands (25%) and in Portugal (45%) during a seasonal influenza due to A(H3N2) virus [1] or in England during the A(H1N1)2009 epidemic (decrease from 43% to 32%) [7]. It is difficult to compare consultation rates and the use of medication with other developed countries, as differences may be due to cultural factors but also to characteristics of healthcare systems and their impact on healthcare seeking behaviour.

Limitations of this study are those common to other retrospective telephone surveys, in particular the refusal of households to respond, the non inclusion of households with mobile phones only, and potential recall bias. As shown in the results, recall bias seems to be limited as the estimated incidence using a 1-week recall period was not significantly different.

A mobile phone-only sample was not included because of its very high cost. This may have resulted in an underrepresentation of young adults and particularly those living alone in urban areas, but this was in part corrected because we used weighting to adjust by age, sex, region and town size for this potential non-coverage bias. On the one hand, an underestimation of the incidence and severity of the disease can not be excluded, because the most affected household members could have been unable to answer the telephone. On the other hand, we collected self-defined flu cases without biological or even practitioner’s confirmation, and therefore other pathogens may have induced flu-like illnesses. We believed, however, that the impact of these possible biases is likely to be limited, our results being consistent with estimates produced by other French data sources.

In order to prevent the spread of the infection in the general population, French public health recommendations for individuals with influenza-like symptoms were centred on the adoption of effective hygiene measures such as covering the mouth and nose with a tissue when coughing and sneezing, performing hand hygiene frequently, cleaning hands immediately after contact with respiratory secretions and wearing a face mask. These recommendations have been widely disseminated in the whole population through television, flyers and internet during the entire study period [10]. These messages seem to have an impact, as more than one third of the population reported that they washed their hands more often this season than during the previous season because of the recommendations that have been made to prevent A(H1N1)2009 transmission (interviews from January to April 2010). A similar increase in handwashing has been observed in other countries in relation to A(H1N1)2009 pandemic, in Hong Kong (30.3%) and England (28.1%) [11,12]. The impact of these recommendations in the general population needs to be taken into account when considering the results for reported handwashing.

Only 11% of the cases used face masks. These results are lower than those reported in studies carried out in France before the pandemic in which 46% and 91% of interviewees declared that they would wear a mask, depending on the type and severity of influenza epidemic [13,14]. In a pandemic situation related to highly pathogenic avian influenza, 96% of interviewees declared in 2006 that they would follow the advice of their physician, and 92% that they would follow the advice of the public authorities [14]. High percentages of adherence to hygiene measure in households are difficult to obtain, even in control studies [15,16]. A study during the SARS crisis indicated that compliance with recommendations reflected anxiety and risk perception [17].

Our study showed that recommendations were better followed by people vaccinated against A(H1N1)2009 influenza, women, children and people living in large towns. This suggests that a higher level of concern about pandemic influenza was observed among these populations. In France, overall A(H1N1)2009 pandemic influenza vaccine uptake was low at 11.1%, although higher vaccination coverage was observed among children [18]. These data suggest that people who felt more concerned about the pandemic were the most able to get vaccinated and to follow the hygiene recommendations. It might be expected that people living in large towns feel at higher risk for influenza because of closer social activities. Other studies also concluded that women are more likely to improve handwashing to prevent the transmission of respiratory disease [11,12,17]. In November 2009, only one third of the French general population considered the A(H1N1)2009 influenza illness to be a “severe” or “very severe disease” [19]. A higher severity of the A(H1N1)2009 epidemic would have certainly led to a better implementation of hygiene measures.

Furthermore, our study showed that despite a wide communication campaign on hygiene measures, one third of influenza cases did not understand the usefulness of face masks. This shows that health education messages should be adapted to provide better explanations. Further educational efforts may help to reduce reluctance to implement hygiene measures and better prepare for future health threats.

## Conclusions

This telephone survey allows a more accurate interpretation of the data derived from healthcare provider-based influenza surveillance systems. GP and hospital-based surveillance systems underestimate the burden of influenza, as around two out of every three cases consulted a GP. Improvement in implementing hygiene measures was observed in the context of the pandemic and the analysis of the determinants gives relevant information for adapting future health education messages.

## Competing interests

The authors declare that they have no competing interests.

## Authors’ contributions

SV, VV, HV, YLS, DLB conceived the study. DV analyzed the results in consultation with SV, VV, HV, YLS, DLB. DV wrote the draft version and revisions of the manuscript according to the contribution of SV, YLS, VV, HV, DLB. All authors read and approved the final version of the manuscript.

## Pre-publication history

The pre-publication history for this paper can be accessed here:

http://www.biomedcentral.com/1471-2458/12/947/prepub
